# Energy and macronutrient intakes in Jordan: a population study

**DOI:** 10.1038/s41598-023-39900-1

**Published:** 2023-08-05

**Authors:** Huda Al Hourani, Buthaina Alkhatib, Islam Al-Shami, Amin N. Olaimat, Murad Al-Holy, Narmeen Jamal Al-Awwad, Mahmoud Abughoush, Nada A. Saleh, Dima AlHalaika, Omar Alboqai, Ayoub Al-Jawaldeh

**Affiliations:** 1 Department of Clinical Nutrition and Dietetics, Faculty of Applied Medical Sciences, The Hashemite University, P.O. Box 330127, Zarqa, 13133 Jordan; 2 Science of Nutrition and Dietetics Program, College of Pharmacy, Al Ain University, 64141 Abu Dhabi, United Arab Emirates; 3 Department of Nutrition and Food Science, Faculty of Agriculture and Sciences, Jerash University, Jerash, Jordan; 4 Regional Office for the Eastern Mediterranean, World Health Organization, Cairo, 7608 Egypt

**Keywords:** Health care, Risk factors

## Abstract

Jordan has never conducted a nutrition survey to determine nutrient and energy intakes. The current study aimed to describe the energy and macronutrient consumed by the Jordanian population. A cross-sectional food consumption study was conducted, including a sample of Jordanians using two non-consecutive 24-h dietary recalls (24-h DR) between October 2021 and March 2022. A total of 2145 males and females aged 8 to 85 years old living in households were studied. The average of two 24-h DRs for each individual was converted into energy and nutrient intakes. After measuring weight, height, and waist circumference, the body mass index (BMI) and waist-to-height ratio (WHtR) were calculated. The percentage of under-reporters was higher in women than men (58.2% vs. 45.9%). Adults and older adult women had the highest prevalence of obesity (29.6%), while adults and older adult men had the highest prevalence of overweight (41.4%). There is a significant increase in energy intake in children, boys, and all adults, compared to the recommended calories. The mean energy percentage (E %) of total fat was 38%, exceeding the upper limit of the Acceptable Macronutrient Distribution Range (AMDR). At the same time, the mean daily dietary fiber intake fell below the recommended levels (ranging from 13.5 g in children to 19.5 g in older adults). The study population consumes more fat and less fiber than the recommended levels. Actions must be taken across all age groups to correct the deviation of energy and macronutrient intakes from the recommended dietary allowances.

## Introduction

Food consumption and dietary habits are being studied extensively around the world; to better understand the role of nutrition in disease prevention and to identify the root causes of public health issues such as obesity, which is a major risk factor for a variety of non-communicable diseases (NCDs) including cardiovascular, kidney disease, type 2 diabetes mellitus, and some cancers^1^. In recent decades, dietary patterns worldwide have changed rapidly and dramatically. The changes are characterized by increased processed food consumption, leading to increased saturated fat and simple sugar consumption^2^. These changes have been linked to overweight and obesity and an increased risk of developing NCDs^3^. In 2016, the global prevalence of overweight and obesity was around 39% and 13%, respectively, of the adult population^4^. Jordan also had a higher prevalence of adult overweight and obesity, with approximately 75% of men and women being overweight or obese^5^.

Food consumption is estimated indirectly through methods such as household food purchases or food balance sheets. Because they provide more food information consumed, data from household and individual food consumption surveys are frequently used to estimate food consumption^6^.

Individual food consumption surveys are quite often preferred because data is gathered at the individual level, allowing for comparisons across age and gender groups; thus, they provide the data required to measure nutrient intakes and their adequacy^7^.

Despite this, Jordan had not previously collected data on individual energy and macronutrient intakes. Surveys describing diet and nutritional status are required to monitor the nutritional status of the population, reveal trends in their health and dietary practices, establish links between food and health, identify age groups at nutritional risk, and develop and implement nutritional policies that are tailored to reflect changes in dietary consumption^8–10^.

Despite the importance of such surveys, some countries conduct them continuously over time, others regularly (annually or biennially), and others irregularly^6^. Furthermore, most countries have undertaken one or more individual-level dietary intake surveys. Some surveys targeted people from infancy to the elderly^11^, while others were only adults^1^.

Most surveys used 24-h dietary recalls (24-h DR) as the primary method for assessing dietary intake, with recall frequencies ranging from one to three, either face-to-face or by phone^3,12^. In addition, a food frequency questionnaire, estimated dietary records, and diet histories were used^1,13,14^.

In Jordan, assessment of dietary intake using the primary method (24-h DR) was used on a small population for assessing specific nutrients. To the best of our knowledge, this will be the first comprehensive study of Jordanians' dietary intakes and anthropometric measurements, serving as a baseline for future regular surveys. As a result, the current study aims to evaluate and present descriptive statistics on energy and macronutrient intakes and identify subgroups at risk of deficient or excessive macronutrient intakes.

## Materials and methods

### Design

A cross-sectional study of Jordan's Population-based Food Consumption Survey (JPFCS) was carried out between October 2021 and March 2022. In this study, a part of the JPFCS's collected data is analyzed and presented.

### Sample size

The sample size was calculated based on the estimated population of the Kingdom by Governorate, Locality, Sex, and Household in 2020^15^. The minimum sample size needed within a margin of error of 5%, and a confidence level of 99% was estimated as 664 households; however, to have higher power, the sample size was expanded to 701 households with a total of 2721 household members were contacted, of which 632 households agreed to participate with 2145 (70.8%) household members.

### Inclusion criteria

All the people aged eight years and older residing in the selected private households consented to participate. The participants were divided into four age groups (8–12, 13–19, 20–64, and 65 years and older).

### Exclusion criteria

Children under eight in the chosen households and pregnant or lactating female subjects were excluded from the study.

### Ethical approval

Individuals who agreed to participate in the survey received detailed information about the objectives; parents were asked for oral approval for their children to participate. No incentive money was offered for taking part in the survey. The Institutional Board Review (IRB) committee at The Hashemite University reviewed and approved the survey protocol (No.7/13/2020/2021). After receiving oral permission from the head of the household, one of the participant's parents signed an informed consent form for children and adolescents, and data collection began in the presence of parents during the first visit. Informed consent was obtained from the other participants of the household. Informed consent was obtained from all subjects and/or their legal guardian(s).

Data were collected by a trained interviewer who has a nutrition background. Each chosen household was only visited once. During the visit, the participants completed a face-to-face questionnaire on sociodemographic data, general health data, and intake of dietary supplements. Weight, height, and waist circumference were measured. Two non-consecutive 24-h DR was also conducted, the first of which was a face-to-face interview, while the second 24-h DR was collected via telephone.

### Dietary assessment

Two non-consecutive 24-h DR obtained dietary intake for all age groups (weekday and weekend). The days of reporting were randomly selected, but participants were able to change them according to their availability for the interview. For children, the 24-h DR was administered in the presence of one of the parents. Participants were asked to recall and state all foods and beverages, including their quantity, preparation method, and most commonly consumed food brand names, from midnight to midnight the previous day. Various portion estimating aids, such as household measurement items and food models, were used; additionally, a well-known idea about the quantity was created by using a colored food atlas containing over one hundred foods and composite recipes consisting of photos of various foods and meals commonly consumed in Jordanian diets—a series of at least four photographs depicting portion sizes for each food item. To calculate the average dietary intake of energy and macronutrients. The consumed food items are linked to the following databases: ESHA's Food Processor®, Nutrition Analysis Software (version 11:0; ESHA Research), Composition of Local Jordanian Food Dishes^16^, and Lebanon Food Composition Data: Traditional Dishes, Arabic Sweets, and Market Foods^17^ were used to analyze the food intake of the two recalls. The average total energy and macronutrient intake for all age groups was calculated.

### Anthropometry

Anthropometric measurements, including height, weight, and waist circumference, were performed in all age groups according to standards by a trained nutritionist. Height was measured to the nearest centimeter, with participants in a standing position with light clothing and barefoot, using a portable wall stadiometer. Body weight was measured to the nearest tenth of a kilogram in the same conditions using a digital scale (Microlife WS 50. Widnau. Switzerland). Waist circumference was measured using an anthropometric tape at the level of the narrowest point between the lower costal border and the iliac crest^18^. Body mass index (BMI)(kg/m^2^) was calculated from the height and weight measurements according to Quetelet's formula^18^: BMI = weight (kg)/height (m^2^) for adults. The Body Mass Index-for-Age Z-Score (BAZ) was calculated for children and adolescents. For the association of BMI-for-age with overweight and obesity, values >  + 1 SD represent overweight, and values >  + 2 SD represent obesity according to WHO reference curves (2007). Values between + 1 SD and − 2 SD were considered normal, and values > − 2 SD were considered thin or underweight^19^. For adults and older adults, BMI classified as < 18.5 kg/m^2^ represents underweight; 18.5–24.9 kg/m^2^ represents normal weight; 25–29.9 kg/m^2^ represents overweight; and when BMI ≥ 30 kg/m^2^, the participant classified as obese^20^.

The waist-to-height ratio (WHtR) was calculated by dividing the 'participant's waist circumference by their height^18^. A WHtR cutoff of ≥ 0.5 is generally accepted as a universal cutoff for obesity in children (aged ≥ 6 years) and adults^21^. However, some subjects in the children, adolescent, and adult groups refused to have their weight and waist circumference measured.

### Misreporting of energy intake

Energy intake (EI) misreporting in self-reported dietary methods, particularly underreporting, is a well-documented phenomenon observed in children and adults. It was observed in all individual self-reported dietary methods using 24 h DR. The basal metabolic rate (BMR) was estimated using the Schofield equations^22^ based on weight, age, and sex. The reported daily EI ratio divided by the estimated BMR was calculated as a measure of the underreporting degree. Participants with EI: BMR ratio of < 1.2 were classified as under-reporters (UnR); EI: BMR of 1.2–2.4 as plausible (PR); and EI: BMR of > 2.4 as over-reporters (OvR) for EI as suggested by Goldberg et al.^23^ and Black^24^.

### Dietary adequacy

Based on the two collected 24-h DR, the average energy and macronutrient intakes of the plausible energy intake reporter participants were calculated and presented. The average daily carbohydrate, protein, and fat intakes were calculated as a percentage of total daily EI and compared to the Acceptable Macronutrient Distribution Ranges (AMDR), which serve as a guideline for the accepted percentage of macronutrient intake^25^. To calculate energy expenditure, BMR was multiplied by the assumed physical activity level (PAL), which was set at 1.55 for all respondents.

### Statistical analysis

Analysis was conducted using SPSS software (IBM SPSS Statistics for Windows, Version 22.0. Armonk, NY: IBM Corp). All analyses were stratified by gender and age group. Sociodemographic and anthropometric characteristics were described using frequencies and percentages for categorical variables. The energy and macronutrient intake distribution was reported as means and standard deviation (SD). A paired samples t-test was used to compare energy intake and expenditure differences. A chi-square (χ^2^) test was performed to examine the differences between categorical variables. The independent sample t-test was used to test the difference in macronutrient intakes among both genders. The normality of the distributions was assessed through the Kolmogorov–Smirnov test and Kurtosis and Skewness values. Differences were considered significant at *p* < 0.05.

### Ethical approval and consent to participate

The study was conducted according to the guidelines of the Declaration of Helsinki. The Institutional Board Review (IRB) committee at The Hashemite University reviewed and approved the survey protocol (No.7/13/2020/2021).

## Results

### General characteristics of JPFCS

A total of 2145 individuals participated in this study, of whom 954 (44.1%) were males, and 1200 (55.9%) were females; their general characteristics are presented in Table 1. Most of the study population were adults; aged 20–64 years (66.6%) and geographically distributed in the central governates in Jordan (62.1%). Nutritional supplements were consumed by approximately 21% of the subjects studied, with Vitamin D supplements having the highest contribution, and the mean family size was 4.5 people in the studied sample. Moreover, nearly half of the sample (49.8%) reported having a household income of less than 500 Jordanian dinars (JOD).

**Table 1:** Characteristics of the population sample stratified by gender.

Study population	Total *n*(%)	Males *n*(%)	Females *n*(%)
	2145 (100.0)	954 (44.1)	1200 (55.9)
Age groups^a^			
Children (8–12 years)	243 (11.3)	124 (13.1)	119 (9.9)
Adolescents (13–19 years)	374 (17.4)	163 (17.2)	211 (17.6)
Adults (20–64 years)	1428 (66.6)	608 (64.3)	820 (68.4)
Older adults (≥ 65 years)	99 (4.6)	50 (5.3)	49 (4.1)
Geographical distribution^a^			
Central governates	1331 (62.1)	573 (60.6)	758 (63.2)
Northern governates	639 (29.8)	287 (30.4)	35(29.3)
Southern governates	175 (8.2)	85 (9.0)	90 (7.5)
Using nutritional supplements^a^			
Yes	448 (20.9)	105 (11.1)	343 (28.6)
No	1697 (79.1)	840(88.9)	857 (71.4)
Family Size^b^	4.5 ± 1.6^a^		
Household Income^a^			
< 500 JD	308 (49.8)		
500–1000 JD	232 (37.5)		
> 1000 JD	79 (12.8)		

^a^*n*(%).

^b^mean ± SD.

In the baseline data of our study, both genders were found to be almost similarly distributed in all other characteristics except dietary supplement use, which was shown to be used by females more frequently than males (28.6% vs. 11.1%) (Table 1).

The anthropometric assessment of the JPFCS study population is shown in Table 2. A significant (*p* =  < 0.001) difference in overweight and obesity status relative to gender. Males were more likely to be overweight than females (42.1% vs. 28.9%, respectively) among adults ,and females were more likely to be obese for the age mentioned earlier than males (29.7% vs. 20.7%, respectively). Moreover, there was a significant (*p* = 0.012) difference in overweight and obesity status among older adults based on gender. Males were more likely to be overweight than females (44.0% vs. 28.6%, respectively), and females were more likely to be obese than males for the same age (53.1% vs. 24.0%, respectively)..On the other hand, males across all of the JPFCS age groups had larger percentages of abnormal ratios in terms of WHtR compared to females in the same age group. Furthermore, the adults category had the greatest abnormal WHtR (66.3% for males and 58.4% for females), which was statistically significant (*p* = 0.002), while the ratio was abnormal in less than 30% of males and around 23% of females in both other age categories.

**Table 2:** Anthropometric assessment of the population sample stratified by age and gender.

Classification	Children		Adolescents		Adults		Older Adults	
	Males	Females	Males	Females	Males	Females	Males	Females
BMI								
Underweight	6 (4.8)	3 (2.5)	30 (18.4)	29 (13.9)	13 (2.1)	30 (3.7)	0 (0.0)	0 (0.0)
Normal	60 (48.4)	66 (55.9)	90 (55.2)	127 (61.1)	213 (35.0)	309 (37.8)	16 (32.0)	9 (18.4)
Overweight	28 (22.6)	32 (27.1)	26 (16.0)	43 (20.7)	256 (42.1)	236 (28.9)	22 (44.0)	14 (28.6)
Obese	30 (24.2)	17 (14.4)	17 (10.4)	9 (4.3)	126 (20.7)	243 (29.7)	12 (24.0)	26 (53.1)
*p*-value	0.309		0.054		** < 0.001**		**0.012**	
WHtR								
Normal	91 (73.4)	90 (76.9)	115 (70.6)	162 (76.8)	205 (33.7)	340 (41.6)	10 (20.0)	8 (16.3)
Abnormal	33 (26.6)	27 (23.1)	48 (29.4)	49 (23.2)	403 (66.3)	477 (58.4)	40 (80.0)	41 (83.7)
*p*-value	0.526		0.173		**0.002**		0.636	

Data are presented as numbers and percent within parenthesis: *n* (%).

WHtR, Waist-to-height ratio.

Significant values are in bold.

### Descriptive analysis of the EI misreporting among the JPFCS study population

As shown in Table 3, 52.8% of JPFCS participants underreported their EI, while only 2.2% overreported; female participants tend to have a higher proportion of EI reporting (58.2%) than males (45.9%), which was significantly different (*p* =  < 0.001). As the JPFCS study population was stratified according to gender and age, both genders tend to under-report their EI and become less reliable when reporting it.

**Table 3:** Under/over reporters of Energy intake among the JPFCS study population stratified by gender.

Age groups	Males			Females		
	UnR	PR	OvR	UnR	PR	OvR
Total	423 (45.9)	471 (51.5)	28 (3.0)	683 (58.2)	474 (40.3)	18 (1.5)
*p*-value	** < 0.001**					
Children	31 (27.2)	76 (66.7)	7 (6.1)	45 (39.1)	68 (59.1)	2 (1.7)
Adolescents	75 (47.8)	80 (51.0)	2 (1.3)	112 (54.4)	89 (43.2)	5 (2.4)
Adults	287 (47.8)	296 (49.3)	18 (3.0)	492 (61.0)	303 (37.6)	11 (1.4)
Older Adults	30 (60.0)	19 (38.0)	1 (2.0)	37 (70.8)	14 (29.2)	0 (0.0)
*p*-value	** < 0.001**			** < 0.001**		

Data are presented as numbers and percent within parenthesis: *n* (%).

JPFCS, The Jordanian Population-based Food Consumption Survey; UnR, Under-reporters; PR, Plausible-reporters; OvR, Over-reporters.

Significant values are in bold.

Although males during adolescence and early adulthood years have the same proportion of being UnR (47.8%), adult females tend to have a higher proportion of being UnR (61%) when compared to UnR adolescent female girls (54.4%). Additionally, older adult males and females have the highest EI reporting percentage (60% and 70.8%, respectively) compared to other age categories. Interestingly, none of the females aged 65 years and older in the JPFCS study population OvR had their EI, and only one male (2%) reported it (Table 3).

### Energy and macronutrient intakes among the JPFCS study population

**Figure 1:**
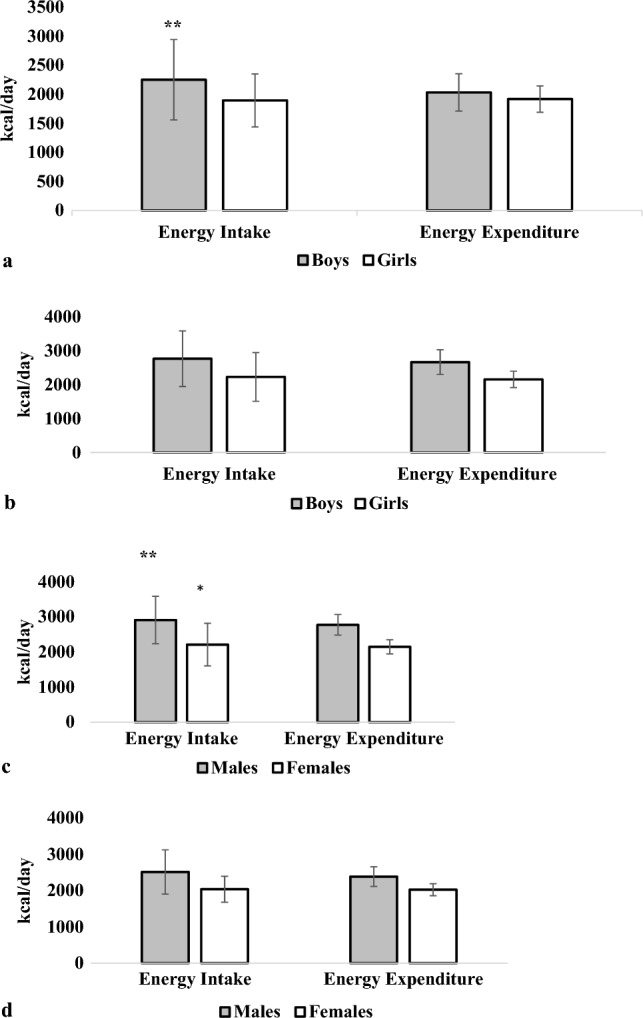
Differences in total daily energy expenditure and energy intake across all age groups: (**a**) for children, (**b**) for adolescents, (**c**) for adults, and (**d**) for older adults. *< 0.05; **< 0.01.

Among the study population, 47.2% were among the plausible EI group. Those 'participants' dietary intakes were included in the analysis to produce our JPFCS nutritional assessment. Table 4 presents the mean daily energy and macronutrient intake consumed by the selected study population. Male adults consumed a higher average amount of energy (2907.5 kcal), followed by adolescent boys (2758.6 kcal) and 'males' older adults (2513.5 kcal). While adolescent girls' mean daily EI is the highest (2220.9 kcal), followed by adult females (2209.3 kcal), female in older adults age has an average EI of (2039.1 kcal). With a *p-*value of 0.05, both genders have a significantly higher average of EI (2757.7 kcal vs. 2614.5 kcal for males and 2161.7 kcal vs. 2096.6 kcal for females) than EE, as shown in Fig. 1, which also displays differences in total daily EE and EI across all age groups. In contrast, the average daily EI of children boys and adult males is significantly higher than their average EE (Fig. 1a and c; *p-*value < 0.05), only the average daily EI of adult females is significantly higher than their average EE (*p*-value = 0.045) (Fig. 1c).

**Table 4:** Mean daily intakes of energy (kilocalories) and macronutrients (grams and percentage contribution to total energy intake) of participants according to age group.

Energy and macronutrients	Children		Adolescents		Adults		Older adults	
	Boys	Girls	Boys	Girls	Males	Females	Males	Females
Total energy, (kcal)	2249.5 ± 691.7	1893.1 ± 455.5***	2758.6 ± 817.3	2220.9 ± 716.5***	2907.5 ± 767.2	2209.3 ± 607.0***	2513.5 ± 609.9	2039.1 ± 357.4*
Total carbohydrates (g)	283.2 ± 99.3	244.2 ± 61.9*	332.4 ± 107.4	282.9 ± 112.0*	345.9 ± 112.1	256.9 ± 72.2***	280.6 ± 112.8	229.4 ± 52.5
% of total energy intake	50.2 ± 7.0	51.6 ± 5.8	48.3 ± 6.6	50.7 ± 6.7	47.7 ± 7.6	46.8 ± 7.2*	44.5 ± 12.6	44.9 ± 5.6
Dietary fiber, g	15.5 ± 9.1	13.8 ± 6.2	18.8 ± 10.2	15.9 ± 7.2*	20.7 ± 8.7	17.8 ± 8.3***	21.9 ± 8.5	18.1 ± 5.6
Dietary fiber, (g/1000 kcal)	6.6 ± 2.2	7.2 ± 2.3	6.7 ± 2.5	7.1 ± 2.4	7.1 ± 2.4	8.0 ± 2.7***	8.9 ± 2.9	8.8 ± 2.3
Total protein (g)	74.5 ± 24.5	61.3 ± 15.3***	100.4 ± 35.6	74.7 ± 25.6***	114.6 ± 37.4	81.8 ± 28.4***	93.8 ± 33.9	82.2 ± 21.9
% of total energy intake	13.4 ± 2.7	13.1 ± 2.4	14.7 ± 3.7	13.6 ± 2.8*	15.8 ± 3.6	14.9 ± 3.4***	15.0 ± 4.0	15.9 ± 2.7
Total fat (g)	94.3 ± 34.1	77.4 ± 25.3*	116.9 ± 41.1	90.5 ± 29.5***	120.9 ± 42.9	97.7 ± 36.5***	117.6 ± 51.8	90.5 ± 19.8
% of total energy intake	37.7 ± 6.0	36.0 ± 5.8	37.9 ± 5.9	36.9 ± 6.2	37.2 ± 7.2	39.4 ± 6.7***	42.1 ± 11.1	40.1 ± 6.1
Water, ml	1679.7 ± 656.8	1570.9 ± 686.5	2152.4 ± 947.5	1997.5 ± 831.3	2844.4 ± 913.1	2602.5 ± 4254.4	3080.2 ± 20.8	2456.0 ± 518.5

Data are presented as means ± SD.

*p < 0.05; ***p < 0.001.

On the other hand, female older adults consumed a higher protein intake (15.9%), followed by male adults (15.8%) and male older adults (15.0%). All age/gender groups' intakes of protein and carbohydrates were within the typical ranges for a balanced diet (45–51% and 13–16%, respectively), while all participants (rather than age group or gender) had a higher tendency to consume a higher percentage of fat exceeded the daily recommendation (36–42%). Regardless of gender, it was apparent that low fiber intake was consumed by children, adolescents, adults, and older adults (14.5 g, 17.5 g, 16.3 g, 19.0 g, and 20.0 g, respectively). Furthermore, the average beverage consumption was the highest among older adult males (3080 ml) and the lowest among children girls (1570 ml) (Table 4).

## Discussion

Individual nutrition surveys for examining food consumption patterns and their relationship to population-level energy, macronutrient, and micronutrient intakes have gained recognition worldwide; however, studies investigating food consumption based on individual nutrition surveys are lacking in the Eastern Mediterranean Region. The current study describes Jordanian children, adolescents, adults, and older adults' energy and macronutrient intakes. To the best of our knowledge, this is the first population-based study that estimates energy, macronutrient, fiber, and beverage intakes among Jordanians and in this region.

The general population's use of dietary supplements has been extensively studied. In the current study, 20.9% of participants (11.1% of males and 28.6% of females) used at least one dietary supplement, and the most commonly used supplements were Vitamin D, multivitamins, and omega 3, respectively. One possible explanation for why vitamin D is the most frequently used supplement is that in Jordan, the prevalence of vitamin D deficiency among males was 54% and 78.5% among females^26^. In another study, multivitamins were reported to be the most commonly used dietary supplements among Jordanian adults, followed by vitamin D and C^27^.

Energy intake is one of two components of energy balance; positive energy balance occurs when EI exceeds EE, which usually results in weight gain, defined as being overweight or obese. Using BMI as an anthropometric index for determining obesity, the current study found that approximately 60% of adults and older adults were overweight or obese, while 37% of children and adolescents were overweight or obese.

Many Jordanian studies reported the prevalence of overweight and obesity across all age groups except for EI. According to a multipurpose national household survey, approximately three-quarters of men and women aged 18–90 were overweight or obese^5^. In a nationwide, cross-sectional study of children aged 6–17 years conducted in 2015, the overall prevalence of overweight and obesity was 17.3% and 15.7%, respectively^28^. The findings of the current study were consistent with previously published studies. Using WHtR as another measure to define obesity, 46% of men and 54% of women were determined to be obese, which was consistent with Ajlouni et al.^5^, who reported that 60.4% of men and 75.6% of women were obese. Even though a cutoff of ≥ 0.5 is widely accepted as a universal level for obesity in children (aged ≥ 6 years) and adults^21^, the WHtR has not been studied extensively in Jordanian children and adolescents. Al Hourani and Alkhatib (2022) reported that 12.2% of children aged 10–14 years in a sample of 793 from the Amman subdistrict had abnormal WHtR^29^.

Energy intake has been extensively studied globally on various age groups using assessment methods with small or national sample sizes. Underreporting is still a problem that should be addressed in surveys designed to estimate EI; in our study, we estimated that 52% of the population was UnR. In the Belgian national food consumption survey on the population aged 15 years and older, 20% of the respondents were UnR^30^. Lundblad et al., 2019 reported a similar proportion in a population-based sample of Norwegians aged 40 to 99 years^1^. Also, similar results were reported in the Australian national nutrition and physical activity survey, though 28.6% of the respondents considered UnR using the Goldberg method, it was 44.5% using the predicted total EE method^31^. In the German national nutrition survey II, the proportion of UnR is 23% for the diet history interviews, 22% for the weighed food records, and 16% for the 24-h DR^32^. Malaysian adult nutrition surveys in 2003 and 2014 reported that the UnR of EI was 53.6% and 61%, respectively^33^.

The proportion of UnR varies widely; this can be attributed to various factors such as dietary assessment methods, female gender, increased age, and obesity^32^. The current study's findings correlate to these factors associated with UnR; furthermore, the first study used a 24-h DR to assess EI in a large population of different age groups, with a large proportion of females.

The current study found a significant increase in EI versus EE in both genders across the entire population, with a significant increase in EI observed in particular among male children and adult males and females. Regardless of these findings, an average of two 24-h DR of EI is not a good predictor of daily EI, particularly in light of the increased prevalence of obesity across all age groups. In addition to the fact that the lockdown during the COVID-19 pandemic also contributed significantly to global obesity rates^34^

Given Jordan's lack of food consumption data, researchers from other countries have reported comparable food consumption and nutrient intake findings. In the current study, for the total population; carbohydrate and protein contributions to EI (48.1% and 15.2%; respectively) were within the AMDR (45–65% of carbohydrate for all age groups and 10–30% of protein for children and adolescent, and 10–35% for adults and older adults). Nonetheless, carbohydrate contribution to EI was lower in older adults than in AMDR (44.5%). The most conspicuous finding was that fat consumption was higher across all age groups than in the AMDR ranges (25–35% for children and adolescents and 20–35% for adults and older adults), exceeding 35% and even 40% in older adults. The contribution of carbohydrates, protein, and fat to EI has been studied at the population level in many countries, particularly developed ones, but to a lesser extent in Middle Eastern countries. The carbohydrate AMDR is characterized by a wide range of 45–65%, and the contributions of total carbohydrates to EI was between 45 and 49% were reported for studies performed in Belgium (45.8%), Norway (42%), Lebanon (48.9%), Italy (45%), and Canada (49.3%)^1,11,12,30,35^. Other studies reported carbohydrate contributions of 50–59% in Malaysia (53%), Brazil (56%), the UK (50%), and the US (50.5%)^2,33,36,37^; and remarkably, (71%) in the Philippines and (66.7% in women) in Mexico^38,39^. The current study's findings are consistent with the Lebanese community^12^; the amount of carbohydrates consumed depends on the type of food habits; in Jordan, three carbohydrate sources contribute to total carbohydrate intake: bread, which is a basal food; rice, which is a primary component of lunch meals; and sugar and desserts.

The 'adult's AMDR of fat ranges between 20 and 35%, with the utmost recommended for less than 30%. The mean contribution of fats to total EI was 38% in all age classes except in older adults (41%). According to surveys conducted in different countries with national samples of adults, the mean proportion of energy from fat in Lebanon (36.9%)^12^, Belgium (37.9%)^30^, Norway (35%)^1^, Italy (36–37%)^11^, UK (35%)^36^, US (32%)^37^, Hungary (36.1–38.9%)^40^, Canada (33.8)^35^, and Malaysia (31%)^33^. Nonetheless, studies in Brazil and the Philippines found that fats contributed 27% and 17% of total EI, respectively^2,38^.

Since the current study included children and adolescents, the contribution of fats to total EI for both age groups was nearly 37%. Some national or population-based studies emphasized fat's average contribution to total energy. In Brazil, the mean contribution of total fat to EI was 27% in the age groups 10–13 and 14–18 years^2^, 36.2% in Belgium^30^, 37% in Italy^11^, and 34.8% in the UK^36^.

The current study reported that the contribution of fat to EI was 41% in older adults (≥ 65 years). According to studies that included the elderly as a subset of the entire population, the Brazilian elderly had the lowest contribution of total fat to total EI (26% for men and 27% for women)^2^, whereas 39–39.9% in older age categories (60–74 and > 75 years) in Belgium^30^, 34% in Italy^11^, and 33.4% in Canada^35^.

The increased contribution of fat to EI in the current study could be attributed to a variety of factors, including changes in food habits during the COVID-19 pandemic lockdown, which were characterized by increased home preparation of foods, particularly traditional sweets, and desserts; reliance on Westernized diets, particularly among children and adolescents; and cooking methods, which could include frying and deep frying. As a result, future work into the fat contents of polyunsaturated, monounsaturated, saturated fat, cholesterol, and trans fat should be conducted.

Protein contributed 15.2% of total EI, within the AMDR for all age groups. Among the reported macronutrient intake data, many studies found the protein within AMDR, whereas carbohydrates and fat were not. In national and population-based studies conducted in Italy, Norway, Canada, the US, the UK, Mexico, Brazil, Belgium, Malaysia, and the Philippines^1,2,11,30,33,35–39^, the protein intake was found to be within AMDR.

The results of the current study were in accord with those of previous studies. In addition to total protein consumption, some studies reported protein consumption by sources, such as animal or plant protein. Conferring to a Brazilian study, more than 50% of the total protein consumed was of animal origin^2^. According to some studies, animal protein consumption among Dutch adults decreased across all age groups in the 2012–2016 national food consumption survey compared to the 2007–2010 survey^3^. Also, the average daily protein intake in the Hungarian population was near 60/40% animal/vegetable protein ratio^40^. The current 'study's result on protein consumption might need further analysis to quantify the ratio of animal to plant proteins.

Although the average daily intake increased with age, ranging from 13.5 g in children to 19.5 g in older adults, fiber consumption was considerably lower than the recommended levels for all age groups, which were set at 14 g/1000 kcal^41^ or more than 25 g /day^42^.

The results of our study are entirely consistent with studies in other countries, which also reported inadequate dietary fiber intake, Slovenia^43^, Brazil^2^, the Philippines^38^, Norway^1^, Spain^44^, Finland^45^, and Hungary^40^. Even though fiber intake was inadequate in all age groups, the current study found that fiber intake increased with age; this finding is consistent with a previous Italian study on a population (0.1–97.7 years), which reported that the mean daily intake of dietary fiber increased with age and ranged from 8 g per day in infants to 22 g per day in the elderly^11^. These findings appear to be possible because children and adolescents consume more fast and refined foods, whereas the elderly prepare more meals at home from raw fruits, vegetables, and grains, which contain more dietary fiber.

In surveys seeking information on EI, beverage consumption must be assessed. A limited number of studies estimated beverage consumption, which included water, coffee/tea, and soft drinks (sweetened or unsweetened). The average total beverage intake in the Spanish study was 850 g/day, with water and sugary soft drinks contributing 569 and 88 g/day, respectively^46^. According to the Danish study, individuals below the lowest quartile of the diet quality index consumed more sugar-sweetened soft drinks, while those above the highest quartile consumed more water^47^. According to another study, beverages, particularly sugary beverages, account for 17.1% of total energy consumption in Brazil^48^. Further analysis of the current study data on beverage consumption is required to categorize it into different types, such as water, tea/coffee, sugary soft drinks, and others.

The current study's findings can, at least partly, explain the role of dietary factors in the increased prevalence of overweight across all age groups and the progression of chronic non-communicable diseases in Jordan. Furthermore, this study was conducted following the COVID-19 pandemic and its effects on food habits and nutrient intake. The study's dietary profile can help support initiatives to lower the prevalence of obesity and diabetes mellitus.

## Conclusion

Our findings provided important insights into the dietary patterns of the Jordanian population, revealing some nutrient deficiencies as well as excessive nutrient intake. It is also necessary to reduce fat consumption while increasing fiber consumption. More efforts are needed to improve the diets of older children and adolescents. It will also be necessary to monitor its progression.

Future research is required in a variety of areas, such as dietary habits for age groups that are at risk, like pregnant women and young children, the connection between energy and macronutrient intakes and obesity across all age groups, but particularly in children and adolescents, and the need to conduct regular surveys of food consumption to track changes over time.

### Strength and limitations

The current study has several strengths: first, it is noteworthy that this is the first population-based study designed to estimate dietary consumption in Jordan. Second, a large population sample of 2145 people aged 8 to 85 years old was recruited; third, rather than self-reporting, participants' heights, body weights, and waist circumference were measured, resulting in much more accurate assessments of BMI and WHtR.;

The study has some limitations that should be considered: the fact that some of the participants were under reporters is a general limitation of all dietary assessment methods, including the current study. Data on physical activity were not obtained. Because the study was cross-sectional, establishing causality between the variables studied was difficult. Another potential limitation is that no biological samples, such as blood or urine, were collected, and no blood pressure was measured.

## Data Availability

Raw data sets are available on request from the corresponding author.

## References

[1] Lundblad, M. W., Andersen, L. F., Jacobsen, B. K., Carlsen, M. H., Hjartaker, A., Grimsgaard, S. *et al*. Energy and nutrient intakes in relation to National Nutrition Recommendations in a Norwegian population-based sample: The Tromso study 2015–16. *Food Nutr. Res.* 63 (2019). 10.29219/fnr.v63.3616 PMC7007761 32082100

[2] Souza RA, Yokoo EM, Sichieri R, Pereira RA (2015). Energy and macronutrient intakes in Brazil: Results of the first nationwide individual dietary survey. Public Health Nutr..

[3] Dinnissen CS, Ocke MC, Buurma-Rethans EJM, van Rossum CTM (2021). Dietary changes among adults in The Netherlands in the period 2007–2010 and 2012–2016. Results from two cross-sectional national food consumption surveys. Nutrients.

[4] World Health Organization. Obesity and Overweihgt Geneva, Switzerland 2021 [Available from: https://www.who.int/news-room/fact-sheets/detail/obesity-and-overweight.

[5] Ajlouni K, Khader Y, Batieha A, Jaddou H, El-Khateeb M (2020). An alarmingly high and increasing prevalence of obesity in Jordan. Epidemiol. Health.

[6] Huybrechts I, Aglago EK, Mullee A, De Keyzer W, Leclercq C, Allemand P (2017). Global comparison of national individual food consumption surveys as a basis for health research and integration in national health surveillance programmes. Proc. Nutr. Soc..

[7] Micha R, Coates J, Leclercq C, Charrondiere UR, Mozaffarian D (2018). Global dietary surveillance: Data gaps and challenges. Food Nutr. Bull..

[8] Shim JS, Oh K, Kim HC (2014). Dietary assessment methods in epidemiologic studies. Epidemiol. Health..

[9] de Ridder D, Kroese F, Evers C, Adriaanse M, Gillebaart M (2017). Healthy diet: Health impact, prevalence, correlates, and interventions. Psychol. Health.

[10] Naska A, Lagiou A, Lagiou P (2017). Dietary assessment methods in epidemiological research: Current state of the art and future prospects. F1000Res.

[11] Sette S, Le Donne C, Piccinelli R, Arcella D, Turrini A, Leclercq C (2011). The third Italian National Food Consumption Survey, INRAN-SCAI 2005–06–part 1: Nutrient intakes in Italy. Nutr. Metab. Cardiovasc. Dis..

[12] Nasreddine L, Ayoub JJ, Hachem F, Tabbara J, Sibai AM, Hwalla N (2019). Differences in dietary intakes among lebanese adults over a decade: Results from two national surveys 1997–2008/2009. Nutrients.

[13] Leclercq C, Arcella D, Piccinelli R, Sette S, Le Donne C, Turrini A (2009). The Italian National Food Consumption Survey INRAN-SCAI 2005–06: Main results in terms of food consumption. Public Health Nutr..

[14] Heuer T, Krems C, Moon K, Brombach C, Hoffmann I (2015). Food consumption of adults in Germany: Results of the German National Nutrition Survey II based on diet history interviews. Br. J. Nutr..

[15] Department of Statistics. Estimated Population of the Kingdom by Governorate, Locality Sex and Households Jordan: Department of Statistics.; 2020 [Available from: http://dosweb.dos.gov.jo/DataBank/Population_Estimares/Municipalities2020.pdf.

[16] Takruri, H., Al-Ismail, K., Tayyem, R. & Al-Dabas, M. Composition of Local Jordanian Food Dishes2020.

[17] Food Composition Data. 2021. Traditional Dishes, Arabic Sweets and Market Foods. [Available from: https://ul.edu.lb/files/ann/20210422-LU-RePa-Report.pdf.

[18] Nieman, D. in *Nutriitional Assessment*. 7 ed: (McGraw-Hill, Education, New York, 2019).

[19] de Onis M, Onyango AW, Borghi E, Siyam A, Nishida C, Siekmann J (2007). Development of a WHO growth reference for school-aged children and adolescents. Bull. World Health Organ..

[20] Obesity: Preventing and managing the global epidemic. (Geneva, Switzerland; 2000). Report No.: 894. 11234459

[21] Yoo EG (2016). Waist-to-height ratio as a screening tool for obesity and cardiometabolic risk. Korean J. Pediatr..

[22] Schofield WN (1985). Predicting basal metabolic rate, new standards and review of previous work. Hum. Nutr. Clin. Nutr..

[23] Goldberg GR, Black AE, Jebb SA, Cole TJ, Murgatroyd PR, Coward WA (1991). Critical evaluation of energy intake data using fundamental principles of energy physiology: 1. Derivation of cut-off limits to identify under-recording. Eur. .J Clin. Nutr..

[24] Black AE (2000). Critical evaluation of energy intake using the Goldberg cut-off for energy intake: Basal metabolic rate. A practical guide to its calculation, use and limitations. Int. J. Obes. Relat. Metab. Disord..

[25] Institute of Medicine (IOM). Dietary Reference Intakes: Applications in Dietary Assessment. (Washington, DC, 2003).

[26] El-Khateeb M, Khader Y, Batieha A, Jaddou H, Hyassat D, Khawaja N (2019). Vitamin D deficiency and associated factors in Jordan. SAGE Open Med..

[27] Basheer A, Elsalem L, Jaber D, Ibraheem S, Alhamad H, Jum'ah A (2021). Knowledge, awareness and practices regarding dietary supplements in Jordan. Trop. J. Pharm. Res..

[28] Zayed AA, Beano AM, Haddadin FI, Radwan SS, Allauzy SA, Alkhayyat MM (2016). Prevalence of short stature, underweight, overweight, and obesity among school children in Jordan. BMC Public Health.

[29] Al Hourani HM, Alkhatib B (2022). Anthropometric indices of obesity as predictors of high blood pressure among school children. Clin. Exp. Hypertens..

[30] Temme E, Huybrechts I, Vandevijvere S, De Henauw S, Leveque A, Kornitzer M (2010). Energy and macronutrient intakes in Belgium: Results from the first National Food Consumption Survey. Br J Nutr..

[31] Goode JP, Smith KJ, Kilpatrick M, Breslin M, Oddy WH, Dwyer T (2021). Retrospectively estimating energy intake and misreporting from a qualitative food frequency questionnaire: An example using australian cohort and national survey data. Front Nutr..

[32] Strassburg A, Eisinger-Watzl M, Krems C, Roth A, Hoffmann I (2019). Comparison of food consumption and nutrient intake assessed with three dietary assessment methods: Results of the German National Nutrition Survey II. Eur. J. Nutr..

[33] Zainuddin AA, Nor NM, Yusof SM, Irawati A, Ibrahim N, Aris T, Huat FL (2019). Changes in energy and nutrient intakes among Malaysian adults: Findings from the Malaysian Adult Nutrition Survey (MANS) 2003 and 2014. Malays. J. Nutr..

[34] Restrepo BJ (2022). Obesity prevalence among U.S. adults during the COVID-19 pandemic. Am. J. Prev. Med..

[35] Ahmed M, Praneet Ng A, L'Abbe MR (2021). Nutrient intakes of Canadian adults: Results from the Canadian Community Health Survey (CCHS)-2015 public use microdata file. Am. J. Clin. Nutr..

[36] Whitton C, Nicholson SK, Roberts C, Prynne CJ, Pot GK, Olson A (2011). National diet and nutrition survey: UK food consumption and nutrient intakes from the first year of the rolling programme and comparisons with previous surveys. Br. .J Nutr..

[37] Shan Z, Rehm CD, Rogers G, Ruan M, Wang DD, Hu FB (2019). Trends in dietary carbohydrate, protein, and fat intake and diet quality among US adults, 1999–2016. JAMA.

[38] Angeles-Agdeppa I, Sun Y, Denney L, Tanda KV, Octavio RAD, Carriquiry A (2019). Food sources, energy and nutrient intakes of adults: 2013 Philippines National Nutrition Survey. Nutr. J..

[39] Barquera S, Rivera JA, Espinosa-Montero J, Safdie M, Campirano F, Monterrubio EA (2003). Energy and nutrient consumption in Mexican women 12–49 years of age: Analysis of the National Nutrition Survey 1999. Salud Publica Mex..

[40] Szeitz-Szabóa M, Bírób L, Bíróa G, Salia J (2011). Dietary survey in Hungary, 2009. Part I. Macronutrients, alcohol, caffeine, fibre. Acta Aliment..

[41] Services. USDoAaUSDoHaH. Dietary Guidelines for Americans, 2020–2025 2020 [9:[Available from: https://www.dietaryguidelines.gov/sites/default/files/2020-12/Dietary_Guidelines_for_Americans_2020-2025.pdf.

[42] Joint WHO/FAO Expert Consultation. Nutrition and the Prevention of Chronic Diseases. Geneva, Switzerland; 2002. Contract No.: (WHO technical report series, 916).

[43] Seljak BK, Valencic E, Hristov H, Hribar M, Lavrisa Z, Kusar A (2021). Inadequate intake of dietary fibre in adolescents, adults, and elderlies: Results of Slovenian representative SI. Menu Study Nutr..

[44] Ruiz E, Avila JM, Valero T, Del Pozo S, Rodriguez P, Aranceta-Bartrina J (2016). Macronutrient distribution and dietary sources in the Spanish population: Findings from the ANIBES study. Nutrients.

[45] Pietinen P, Paturi M, Reinivuo H, Tapanainen H, Valsta LM (2010). FINDIET 2007 Survey: Energy and nutrient intakes. Public Health Nutr..

[46] Partearroyo T, Samaniego-Vaesken ML, Ruiz E, Aranceta-Bartrina J, Gil A, Gonzalez-Gross M (2019). Current food consumption amongst the Spanish ANIBES study population. Nutrients.

[47] Knudsen VK, Fagt S, Trolle E, Matthiessen J, Groth MV, Biltoft-Jensen A (2012). Evaluation of dietary intake in Danish adults by means of an index based on food-based dietary guidelines. Food Nutr. Res..

[48] Pereira RA, Souza AM, Duffey KJ, Sichieri R, Popkin BM (2015). Beverage consumption in Brazil: Results from the first National dietary survey. Public Health Nutr..

